# Supplementary material for: Glia Cells Control Olfactory Neurogenesis by Fine-Tuning CXCL12
Source: Cells. 2023 Aug 28;12(17):2164. doi: 10.3390/cells12172164 (PMC10486585; doi:10.3390/cells12172164)
Supplement: Supplementary file 1 [file cells-12-02164-s001.zip › cells-2573660-supplementary.pdf]

Glia Cells Control Olfactory Neurogenesis by Fine-Tuning CXCL12

André Dietz †, Katja Senf †, Julia Karius, Ralf Stumm and Eva Maria Neuhaus

Supplementary Table S1. Primary antibodies

primary antibody	host species	Dilution	company (order number)	Antibody characterization
ACKR3	mouse	1:30	R&D Systems MAB42273	Absence of ACKR3 staining in *Ackr3*-deficient OE (this study)
KRT5	rabbit	1:500	Convance (PRB-160P)	raised against a peptide sequence derived from the C-terminus of the mouse keratin 5 protein, used to stain horizontal basal cells in the OE, e.g.{Suzuki, 2015 #1713}
CXCL12	rabbit	1:200	Torrey Pines Biolabs (TP-202)	Purified antibody, reactivity against mouse and rat (manufacturer information), staining was abolished by peptide block and overlaps with RFP staining in CXCL12-RFP mice {Senf, 2021 #1730}
CXCR4 (2B11, N-term)	rat	1:500	Affymetrix (14-9991-82)	recognizes the amino terminus of mouse CXCR4, labeling absent in CXCR4 KO animals {Senf, 2021 #1730}
CXCR4 (C-term; UMB-2)	rabbit	1:300	self-made	Raised residues 338–359 of the human CXCR4 receptor, labeling absent in Cxcr4 deficient mice {Fischer, 2008 #748}, labeling absent in OE from E18 *Cxcr4* KO mice. {Senf, 2021 #1730}
KRT8	rat	1:100	DSHB TROMA-I-c	TROMA-I specificity for keratin K8 has been knock-out validated. See PMID: 27234655.
Gamma-tubulin	rabbit	1:500	Sigma, T3559	Staining of gamma-tubulin (48 kDa) by immunoblotting is specifically inhibited with gamma-tubulin peptide (human, amino acids 38-53 with C-terminally added lysine).

GAP43	mouse	1:500	Merck Millipore (MAB347)	immunizing agent: GAP-43 purified from rat brain) recognizes GAP-43 regardless of its phosphorylation state. Immunoblot of membrane fractions of growing neurons reveals an anomalous behavior in sodium dodecyl sulfate (SDS) gels such that its reported molecular mass ranges from 43 to 57 kDa, whereas the primary sequence of the protein indicates a molecular mass of about 24 kDa (manufacturer information).
GM-130	mouse	1:100	BD Biosciences (610822)	Immunofluorescence (Marra et al. 2001)
HA-tag	rabbit	1:800	Cell Signaling (C29F4)	HA-Tag (C29F4) Rabbit mAb detects exogenously expressed proteins containing the HA epitope tag. The antibody may cross-react with a protein of unknown origin ~100kDa.
Heparan sulfate	mouse	1:100	AMSBIO (370255-S)	The antibody reacts with an epitope present in many type of heparan sulfate. The epitope includes (an) N-sulfated glucosamine residue(s) that are critical for the reactivity of the antibody. The antibody does not react with hyaluronan, chondroitin sulfate, dermatan sulfate, keratan sulfate or DNA.
MKi67	rabbit	1:200	Abcam (ab16667)	Knock-out validated due to manufacturer information
MCM2 (BM-28)	mouse	1:200	BD Biosciences (610700)	Raised against human BM28 aa. 725-888, reactive to mouse protein (manufacturer)
ICAM1	goat	1:200	R&D Systems AF796	Detects mouse ICAM-1 in direct ELISAs and Western blots and immunofluorescence. 48 publications on manufacturer website.
OMP	goat	1:500	Wako (544-10001)	Multiple immunizations of rodent olfactory marker protein, widely used to stain olfactory neurons, recapitulates the canonical staining pattern for OMP described for the goat anti-OMP originally distributed by Frank Margolis {Keller, 1975 #1710}, extensively characterized previously{Rodriguez-Gil, 2008 #1711}

Syndecan-1 (CD138)	rat	1:100	BD Pharmingen (553712)	The 281-2 monoclonal antibody specifically binds to the core protein of CD138 (Syndecan-1), a cell-surface, integral membrane heparan sulfate- and chondroitin sulfate-containing proteoglycan that binds to interstitial extracellular matrix molecules.
pS6	rabbit	1:100	Cell Signaling (2215S)	Phospho-S6 Ribosomal Protein (Ser240/244) Antibody detects endogenous levels of ribosomal protein S6 only when phosphorylated at serines 240 and 244. This antibody does not detect S6 ribosomal protein phosphorylated at other sites.
Tomato	goat	1:200	Sicgen (AB8181)	Goat polyclonal antibody to tdTomato (red fluorescent protein), detects tdTomato in brain sections by IHC and WB, does not cross-react with GFP (manufacturer information), used in 48 publications according to manufacturer homepage, also in nervous system, e.g.{Laukoter, 2020 #1709}, no staining in OE of wt mice (Fig. S1A)
MARCKS	rabbit	1:50	Proteintech (20661-1-AP)	20661-1-AP targets MARCKS in WB, IP, IHC, IF, FC, ELISA applications and shows reactivity with human, mouse, rat samples.
LSD1	rabbit	1:500	Abcam (ab129195)	Knock-out validated due to manufacturer information

Supplementary Table S2. Secondary Antibodies

secondary antibody	company (order number)
Alexa Fluor® 488 donkey anti-goat	Thermo Fisher Scientific (A-11055)
Alexa Fluor® 488 donkey anti-mouse	Thermo Fisher Scientific (A-21202)
Alexa Fluor® 488 donkey anti-rabbit	Thermo Fisher Scientific (A-21206)
Alexa Fluor® 488 donkey anti-rat	Thermo Fisher Scientific (A-21208)
Alexa Fluor® 568 donkey anti-goat	Thermo Fisher Scientific (A-11057)
Alexa Fluor® 568 donkey anti-mouse	Thermo Fisher Scientific (A-10037)
Alexa Fluor® 568 donkey anti-rabbit	Thermo Fisher Scientific (A-10042)
Rhodamine Red ™-X donkey anti-rat	Jackson Immuno Research (706-295-148)
Alexa Fluor® 633 donkey anti-goat	Thermo Fisher Scientific (A-21094)
Alexa Fluor® 647 donkey anti-rabbit	Thermo Fisher Scientific (A-31573)
Alexa Fluor® 647 donkey anti-rat	Jackson Immuno Research (712-605-153)

Supplementary Table S3

Genotype	Biological effect of the altered genotype	CXCR4 activation	Outcome
Tg(Mpz-Cre); *Ackr3*$^{loxP;lopP}$	Reduced/no ACKR3 expression in sustentacular cells	CXCR4 over-activation and downregulation	reduced stem cell proliferation, decreased number of immature neurons, increased number of mature neurons
ACKR3-ST/A (homozygous knock-in mice)	Expression of scavenging defective ACKR3 instead of normal ACKR3	CXCR4 over-activation and downregulation	reduced stem cell proliferation, decreased number of immature neurons, increased number of mature neurons
Tg(Mpz-Cre); *Cxcl12*$^{loxP;loxP}$	Reduced/no CXCL12 expression in sustentacular cells	Reduced CXCR4 activation, increased CXCR4 localization on plasma membrane	Increased number of immature neurons, slightly reduced number of mature neurons
Tg(Krt14-Cre); *Cxcl12*^{loxP}	Reduced/no CXCL12 expression in HBCs	No effect showing that *Cxcl12* expression in HBC does not play a role for CXCR4 activation	Similar to WT
Idua-/- (homozygous knock-out of *Idua*)	More HS in the extracellular matrix, more CXCL12 on HBCs	CXCR4 over-activation and downregulation	reduced stem cell proliferation, decreased number of immature neurons, increased number of mature neurons
HA-ACKR3	Expression of HA-tagged ACKR3 instead of normal ACKR3	No effect (data not shown)	Similar to WT (data not shown)
Ackr3-GFP	Expression of GFP und control of the *Ackr3* promoter, allows to identify Ackr3 expressiong cells	No effect (data not shown)	Similar to WT (data not shown)
CXCL12-RFP	Expression of RFP-tagged CXCL12, overexpression of CXCL12	CXCR4 over-activation and downregulation [16]	reduced stem cell proliferation, decreased number of immature neurons, increased number of mature neurons [16]

Supplementary Figures

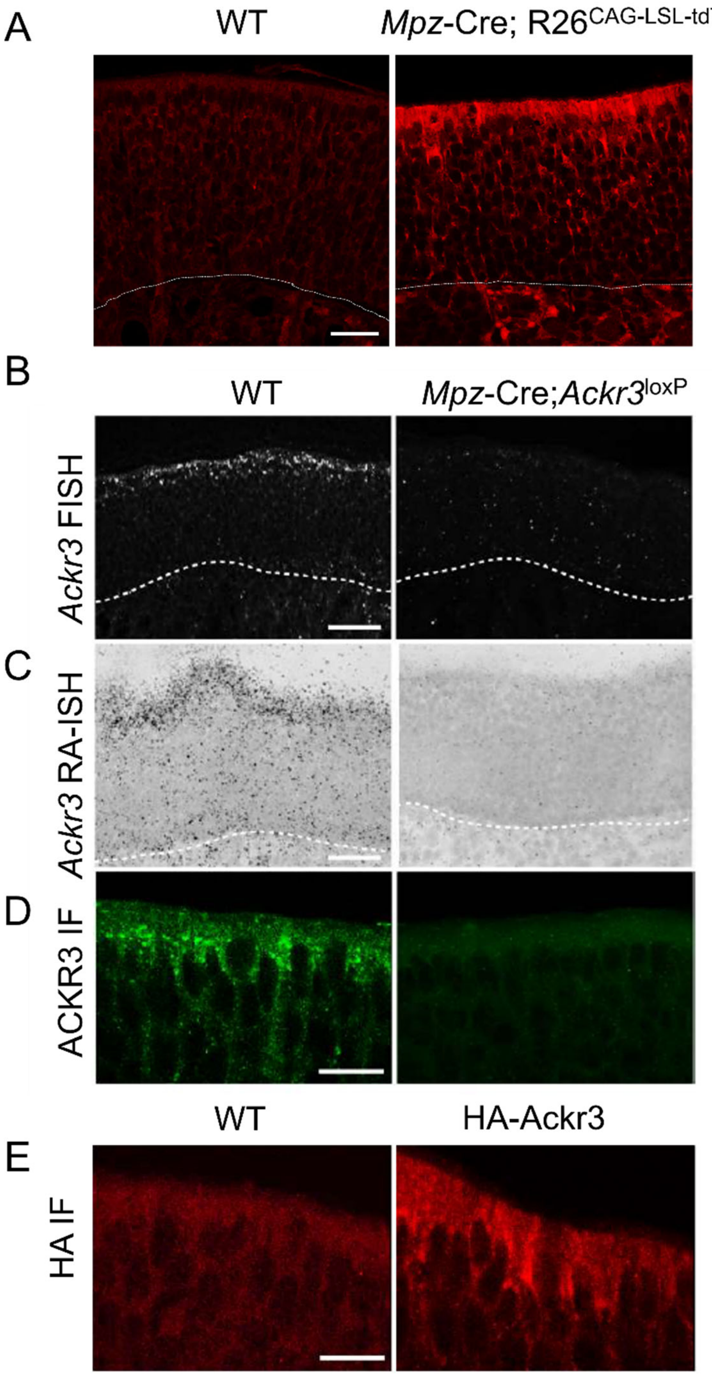

Supplementary Figure S1. Generation of *Ackr3* knock-out in sustentacular cells of the OE. (**A**) TdTomato immunofluorescence in the OE of WT and *Mpz*-Cre;R26$^{\mathrm{CAG\text{-}LSL\text{-}tdT}}$ mice (**B**) Confocal microscopy of the OE of P8 mice after *in-situ* hybridization with digoxigenin-labeled probe (FISH), (**C**) radioactive in-situ hybridization of *Ackr3* (RA-ISH) and (**D**) ACKR3 immunostaining in *Mpz*-Cre;*Ackr3*$^{\mathrm{LoxP/LoxP}}$ mice, all showing downregulation of *Ackr3*. Immunofluorescence staining of *Mpz*-Cre;*Ackr3*$^{\mathrm{LoxP/LoxP}}$ mice showing downregulation of the protein and specificity of the ACKR3 antibody.

(**E**) Staining of WT and HA-ACKR3 mice with anti-HA antibody showing specificity of the HA staining. Scale bars 50 µm (A, B, C); 5 µm (D, E).

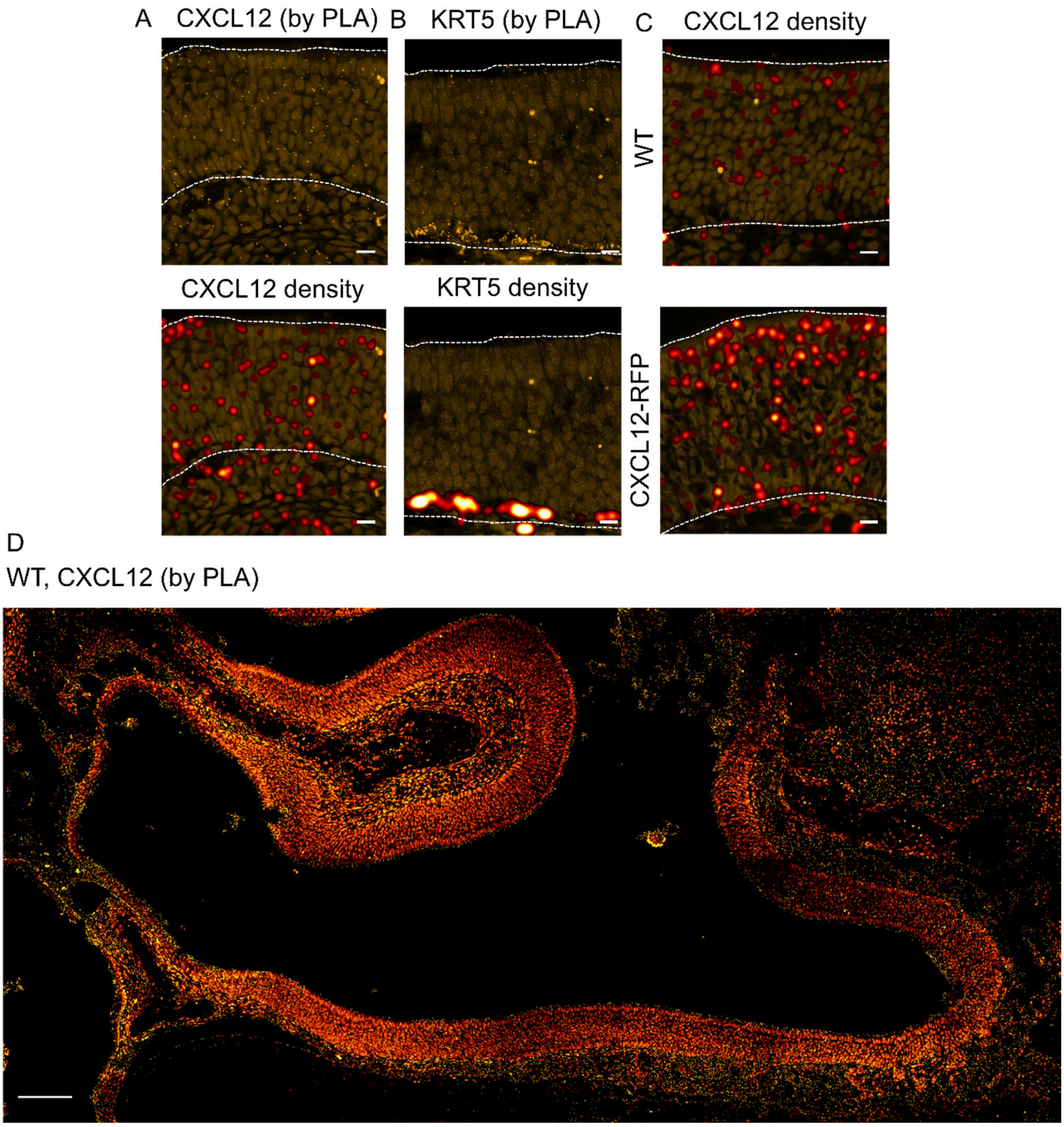

Supplementary Figure S2. Control of PLA experiments. (**A**) OE section stained for CXCL12 (by PLA) as well as CXCL12 density calculated from PLA signal. Adjacent sections stained for CXCL12 by IF show normal labeling of HBCs (not shown). (**B**) Staining for KRT5 (by PLA) as well as CXCL12 density calculated from PLA signal. KRT5 as intracellular protein can readily be detected in HBCs. **(C**) CXCL12 density calculated from PLA signals from WT and CXCL12-RFP animals overexpressing CXCL12 (P8). (**D**) Tile scan of OE section (WT) stained for CXCL12 (by PLA). Scale bars 10 µm (A, B, C), 100 µm (D).

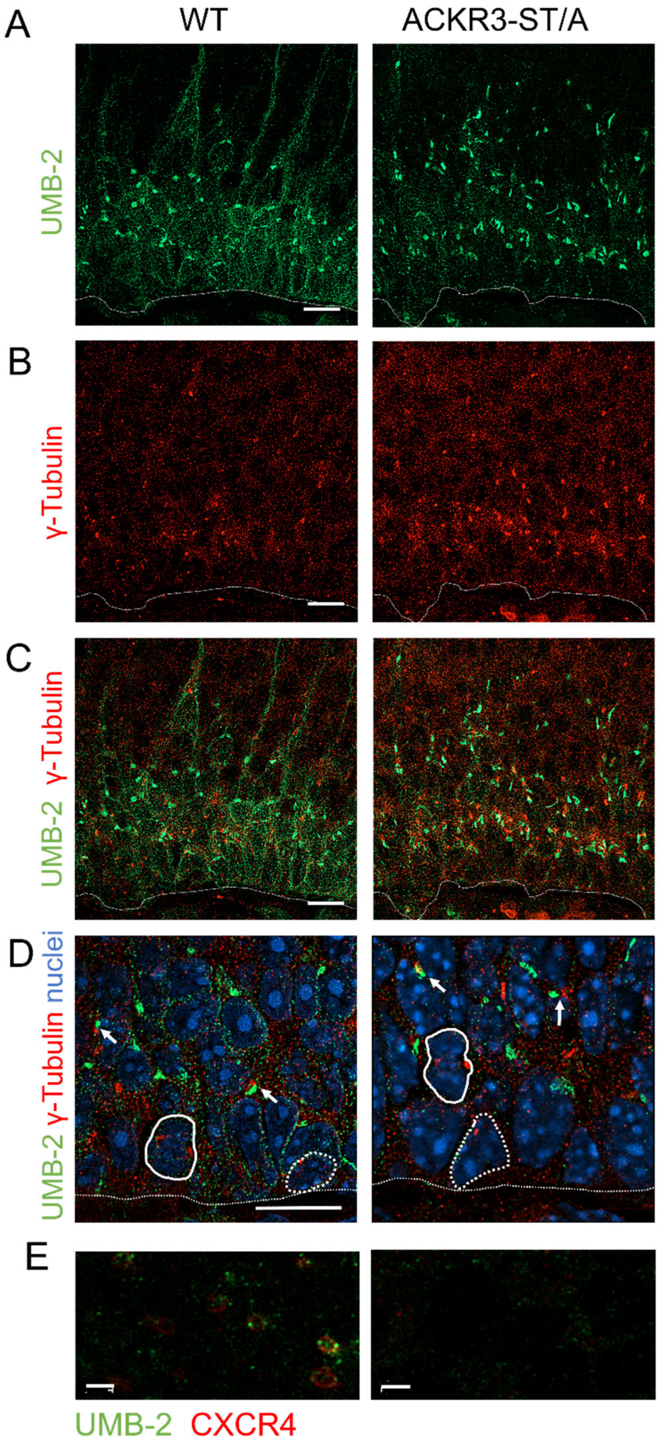

Supplementary Figure S3. Co-localization of CXCR4 with γ-tubulin. Immunolabeling of (**A**) non-phosphorylated CXCR4 (UMB-2), green, (**B**) γ-tubulin as marker for the MTOC, centrosomes and ciliary basal body (red) and (**C**) overlay of both stainings in the OE of WT and ACKR3-ST/A mice (P8). Staining intensity of γ-tubulin is enhanced in the OE with CXCR4 over-activation (ACKR3-ST/A). (**D**) High

magnification single confocal sections showing that intracellular CXCR4 (green) is found adjacent to γ-tubulin (red) positive perinuclear structures (nuclei labeled with Hoechst, blue), but stainings did not overlap. CXCR4-positive structure were not found in HBCs (encircled by dotted line), which show γ-tubulin-labeling at the ciliary base. CXCR4-positive structures were moreover not found in dividing GBCs (encircled by solid line), which show γ-tubulin-labeling at the centrosomes. Scale bars 10 µm, dotted lines delineate the basal lamina.

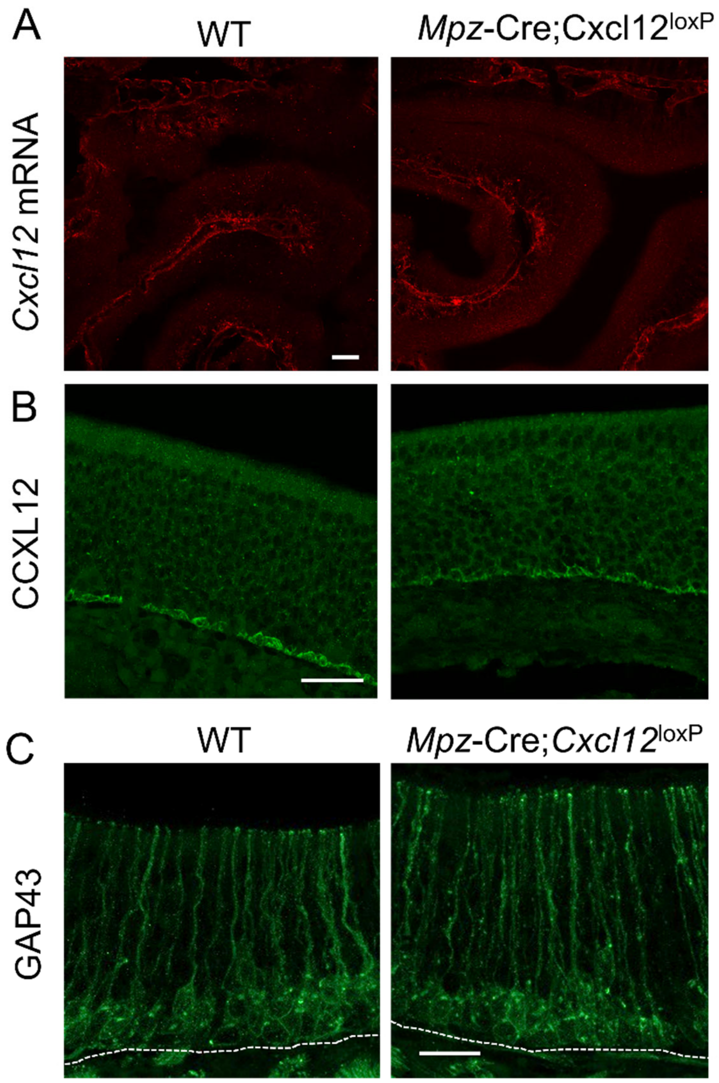

Supplementary Figure S4. Control experiments for Fig. 6 (**A**) Confocal microscopy of the OE of WT and *Mpz*-Cre;*Cxcl12*$^{LoxP/LoxP}$ mice (8W) after *in-situ* hybridization with digoxigenin-labeled probe for *Cxcl12*. *Cxcl12* mRNA expression in the LP is not different between both genotypes, showing that

expression in the LP is not affected in *Mpz*-Cre;*Cxcl12*$^{LoxP/LoxP}$ mice. (**B**) Immunolabeling of CXCL12 in the OE of P8 WT and *Mpz*-Cre;*Cxcl12*$^{LoxP/LoxP}$ mice did not show differences. (**C**) Immunolabeling of GAP43 in the OE of adult (8W) WT and *Mpz*-Cre;*Cxcl12*$^{LoxP/LoxP}$ mice. Labeling of the dendritic membrane and knobs was increased in *Mpz*-Cre;*Cxcl12*$^{LoxP/LoxP}$ compared to WT mice. Shown are projection of confocal section, total thickness of the projection 1 μm. Scale bars 100 μm (A); 50 μm (B); 20 μm (C).

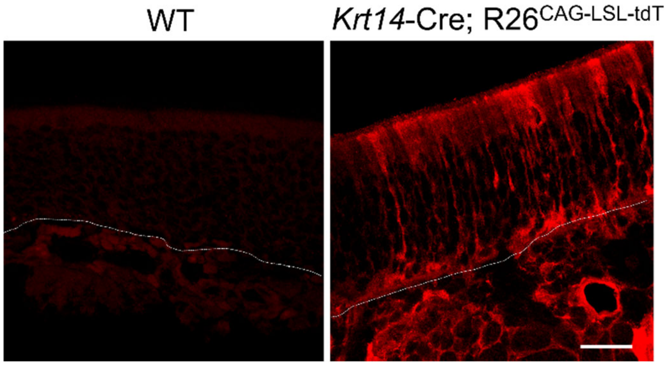

Supplementary Fig 5. TdTomato immunofluorescence in WT and *Krt14*-Cre;R26$^{CAG-LSL-tdT}$. Scale bar 50 μm.
